# Is the MAPK ERK5 the nexus from FAO to NK cell-mediated metastasis immune surveillance?

**DOI:** 10.3389/fimmu.2025.1641865

**Published:** 2025-10-08

**Authors:** Martin Villalba, Delphine Gitenay, Sara Zemiti, Jean-François Rossi, Mauricio Campos-Mora

**Affiliations:** ^1^ IRMB, Univ Montpellier, INSERM, CHU Montpellier, CNRS, Montpellier, France; ^2^ Institut du Cancer Avignon-Provence Sainte Catherine, Avignon, France; ^3^ IRMB, Univ Montpellier, INSERM, CHU Montpellier, Montpellier, France

**Keywords:** Erk5, CD36, fatty acid oxidation (FAO), epithelial-mesenchymal transition (EMT), natural killer (Nk) cell, immune surveillance

## Abstract

Mammalian cells adapt to their environment by reshaping their metabolism. Increased fatty acid oxidation (FAO) enables metastatic cells to enhance their motility and colonize new niches, where the fatty acid transporter CD36 functions as both marker and driver of this process. The MAPK ERK5 regulates CD36 expression, FAO, and the epithelial-mesenchymal transition (EMT), a critical initial step in metastasis. Contrary to popular belief, metastasis is a highly inefficient process, in part due to natural killer (NK) cell immune surveillance. This cytotoxic lymphocyte lineage detects inhibitory and activating ligands on target cells to determine their fate. During EMT, the expression of specific ligands on metastatic cells triggers their recognition by NK cells. Interestingly, several of these ligands are regulated by ERK5. We hypothesize that ERK5 may serve as a central link between FAO, metastasis, and immune surveillance. Here, we review current knowledge and available evidence regarding ERK5 expression in tumor cells and its role in cancer cell migration and metastasis and speculate in the potential role of ERK5 in immune recognition and the clearance of metastasis by NK cells.

## 1 Metastatic cells rely on fatty acid oxidation

Metabolism is altered in cancer cells. Since Otto Warburg described aerobic glycolysis (1) many more metabolic alterations have been uncovered, including the upregulation of mitochondrial respiration alongside glycolysis in certain tumors (2). Thus, cancer cells adapt to changing environment by adjusting their metabolism to new conditions-for example, when they migrate and colonize new tissues. This migration, known as metastasis, is highly inefficient because metastatic cells must overcome numerous obstacles to establish colonies in distant tissues (3). Once metastatic lesions are formed, current treatments often fail to provide lasting responses. During their journey to a new “home”, tumor cells encounter a challenging metabolic environment and respond by increasing fatty acid (FA) consumption and generating energy through FA β-oxidation (FAO) (4). Consequently, lipid oxidation, which fuels β-oxidation and mitochondrial respiration, is linked to tumor progression, invasion and the metastatic process (5–7).

**Figure 1:**
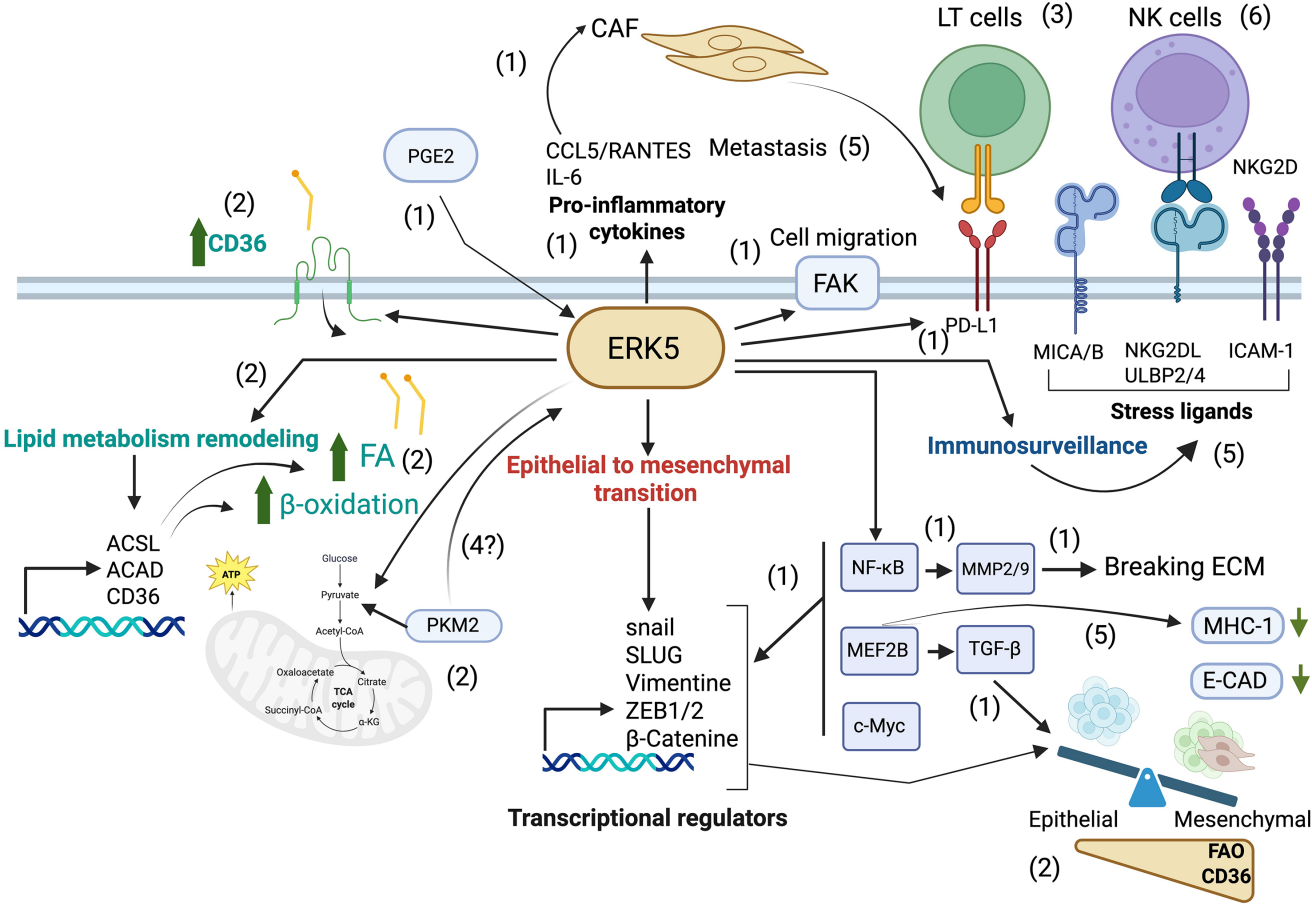
Pivotal role of ERK5 in metastasis. The epithelial-to-mesenchymal transition (EMT) is a critical step in metastasis. ERK5 promotes the accumulation of NF-κB, which activates MMP9, a matrix metalloproteinase that facilitates tumor cell detachment and invasion (1), and phosphorylates FAK favoring tumor cell migration (1). ERK5 also upregulates MEF2B, leading to the activation of TGF-β, a potent EMT inducer (1), and further promotes EMT by activating transcriptional regulators including SNAIL, SLUG, and ZEB/2, resulting in the downregulation of E-cadherin, a hallmark epithelial marker (1). In addition, ERK5 enhances the secretion of pro-inflammatory cytokines such as IL-6 and CCL5, which in turn activate cancer-associated fibroblasts [CAFs; (1)]. After gaining a mesenchymal phenotype, metastatic cells depend on metabolic reprogramming, e.g. to fatty acid β-oxidation [FAO, (2)], to adapt and colonize new environments. ERK5 supports the increased lipid requirements of metastatic cells by promoting fatty acid (FA) accumulation (2) through upregulation of the CD36 receptor (2), and by sustaining FAO (2) via enhanced expression of metabolic enzymes such as ACSLs and ACADs (2), which increases OXPHOS in mitochondria (2). During their journey, metastatic cells can interact with immune cells. By modulating tumor cell metabolism, ERK5 can modulate tumor immune recognition by cytotoxic lymphocytes (3). It is tempting to speculate that mitochondrial changes can signal ERK5 activation through PKM2 favoring tumor cell detachment (4), but also immune recognition by modulating expression of stress ligands (5), ICAM-1 (5) and MHC-I (5). CAFs also contribute to immune evasion by upregulating PD-L1 expression, thereby engaging the PD-1 receptor on T lymphocytes and suppressing their cytotoxic activity (5). By modulating all these molecules, ERK5 regulates NK cell-mediated immune surveillance (6), supporting a central role for ERK5 in the metastatic process. Created in BioRender. Zemiti, S. (2025) https://BioRender.com/.mn9cduu.

Interestingly, to sustain FAO, metastatic cells must increase FA import and activate various genes required for FAO, particularly CD36 (4, 6, 8). Our group has recently shown that this FA transporter is induced by extracellular-regulated kinase 5 (ERK5) (9), also known as big mitogen-activated protein kinase 1 (BMK1) due to its large size (110 kDa), and encoded by the *mitogen-activated protein kinase 7* gene (*MAPK7*). This MAPK plays multiple roles in cancer, including metastasis (10, 11). Tumor cells express higher levels of ERK5 and/or *ERK5* mRNA compared to their normal counterparts (12), and its expression levels may have prognostic value (13, 14). In fact, ERK5 is involved in several of the so-called hallmarks of cancer (10). **Figure 1** summarizes its roles in these biological processes.

## 2 ERK5 regulates FAO

ERK5, described as a stress kinase (15), regulates cell metabolism (16–19), enables cells to manage oxidative stress (19–22), and mediates lipid metabolism in various cellular contexts (9, 23, 24). Knockdown of the MEK5/ERK5 axis in small-cell lung cancer (SCLC) cells not only reduced proliferation and tumor growth but also altered lipid metabolism-related pathways, including those involved in cholesterol homeostasis and FA metabolism (23). Additionally, transcriptomic analysis revealed connections between ERK5 signaling and pyruvate metabolism, as well as the citrate cycle (23). ERK5 enhances intracellular lipid availability by upregulating fatty acid transporters (9), supports FA activation by inducing the expression of long-chain acyl-CoA synthetase (ACSL) enzymes, and sustains FAO through the expression of Acyl-CoA dehydrogenases (ACADs) (9).

The final steps of FAO require oxidative phosphorylation (OXPHOS) via the redox potential in mitochondria. Recent evidence shows that mitochondrial complex I (CI) promotes kidney cancer metastasis (7), consistent with earlier findings that mutations in the ND2 subunit of mitochondrial complex I increase metastatic potential (25). Interestingly, mitochondrial CI also triggers an antioxidant response through ERK5 (19). In a related context, ACSL4 is a pro-hematogenous metastasis factor that facilitates metastatic extravasation by enhancing membrane fluidity and cellular invasiveness (26). Another ACSL family member, ACSL1, also promotes metastasis in endometrial cancer (27). As noted above, ERK5 induces ACSL expression (9). Therefore, ERK5 facilitates transcriptome remodeling to support FAO, which may be linked to pro-metastatic intracellular signaling.

## 3 ERK5 regulates epithelial-to-mesenchymal transition and metastasis

Increasing evidence supports the role of FA incorporation and metabolism in cancer and tumor cell migration (28–31). Consistent with its role in FAO, ERK5 activity enhances cell motility (10, 32), cell migration and invasiveness (32–34), EMT, and metastasis (11, 32, 35, 36). *MAPK7* gene silencing is associated with reduced migration, invasion, and proliferation in osteosarcoma, oral squamous cell carcinoma (OSCC), and prostate cancer cells (37–40). In breast cancer cells, the MEK/ERK5 pathway is essential for suppressing the estrogen receptor, which is necessary to induce EMT and metastasis (41, 42).

Mechanistically, ERK5 activation upregulates focal adhesion kinase (FAK) expression and promotes its activation through direct phosphorylation, thereby facilitating cell migration and metastasis (32, 43–45). Conversely, *MAPK7* knockout in triple-negative breast cancer (TNBC) and prostate cancer cells disrupts the interaction between FAK and proline-rich tyrosine kinase 2 (PYK2), reducing cell attachment to substrates such as vitronectin and fibronectin (46).

In melanoma xenografts, lung and lymph node metastases were drastically reduced upon transfection with dominant-negative isoforms of MEK5 and ERK5 (32). Similarly, *MAPK7* knockout in TNBC cell lines impaired cell migration by diminishing mesenchymal features, including the expression of matrix-associated genes, integrins, and pro-angiogenic factors (47). Pharmacological inhibition of ERK5 reduced prostaglandin E2 (PGE2)-induced proliferation, migration, invasion, and stemness in non-small cell lung cancer (NSCLC) cells, highlighting the critical role of ERK5 in PGE2-induced tumorigenesis (48).

Another essential step in cell invasion is the degradation of extracellular matrix (ECM). In this context, ERK5 enhances the release of ECM metalloproteinases (MMPs), facilitating ECM breakdown and local tumor invasion (47, 49). ERK5 signaling has been shown to increase MMP2 and MMP9 gene expression (37), while ERK5 reduction reduced MMP9 mRNA and protein expression in lung metastases of osteosarcoma (49). A recent study using single-cell RNA sequencing to compare gene expression in primary osteosarcoma and circulating tumor cells (CTCs) supported the importance of the ERK5/MMP9 axis as a key driver of bone tumor metastasis (50). Furthermore, in a xenograft mouse model, *MAPK7* silencing reduced tumor growth and significantly decreased metastatic spread to the lungs, which was associated with reduced *MMP9* mRNA expression and secretion by osteosarcoma cells (50).

In breast cancer cells, overexpression of a constitutively active form of Signal transducer and activator of transcription-3 (STAT3) increased the expression of the EMT markers SNAIL, SLUG and vimentin via the MEK5/ERK5 pathway, while downregulating the epithelial marker E-cadherin (51). Similarly, expression of EMT-related transcription factors such as SNAIL, SLUG, and ZEB1/2 was significantly downregulated in pancreatic cancer xenografts from mice treated with XMD8-92, an ERK5 inhibitor that also reduced tumor growth; although the specificity of this inhibitor for ERK5 remains controversial (52). Other downstream targets of the ERK5 pathway, such as MEF2, activator protein-1 (AP-1), c-Myc (33) and NF-κB (53, 54), play critical roles in regulating EMT transcription factors like SNAIL, SLUG, and β-catenin (33, 55). Notably, MEF2B silencing prevents TGFβ-dependent EMT induction in murine epithelial mammary gland cells (11). In colon cancer cells, constitutive ERK5 activation results in nuclear accumulation of NF-κB, which upregulates vimentin expression and enhances cell migration (56). Accordingly, treatment with the MEK5/ERK5 inhibitor XMD8–92 decreases NF-κB transcriptional activity and reduces the expression of pluripotency transcription factors in colon cancer cells (57). Taken together, these findings confirm the direct and indirect roles of ERK5 in inducing EMT and metastasis across various malignancies, underscoring the potential of targeting the MEK5/ERK5 signaling pathway to develop more effective therapies aimed at preventing or limiting tumor metastasis.

## 4 Tumor cell metabolism modulates recognition by immune cells

Although “avoiding immune destruction” and “deregulating cellular energetics” were not initially recognized as hallmarks of cancer (58), they were later included in subsequent discussions (59). In the meantime, the link between tumor metabolism and immune escape became well established (18), and the potential for using metabolism-modulating drugs to enhance immunotherapy was proposed (60). Currently, there is significant interest in uncovering the mechanisms through which changes in tumor metabolism contribute to immune escape (61), with the aim of developing new immunotherapies (62). This topic has been extensively reviewed in the literature, e.g., (63, 64), and will not be further discussed here.

## 5 Metastatic metabolism and immune recognition

This issue was recently highlighted in an editorial (65). Interestingly, it was noted that pyruvate kinase isoform M2 (PKM2), a key rate-limiting enzyme catalyzing the conversion of phosphoenolpyruvate to pyruvate, plays a significant role in metastasis (66), partially via the ERK1/2 pathway (67). In that study, the authors did not explore whether PKM2 could also modulate ERK5 activity, which is essential for oxidative phosphorylation (OXPHOS) and the antioxidant response (19, 21, 68). Pyruvate produced by PKM2 is consumed in the mitochondria by mitochondrial CI, which regulates ERK5 activity through fumarate accumulation (19). Given that ERK5 promotes EMT and metastasis (as discussed above), it is tempting to hypothesize that PKM2 contributes to metastasis through CI and ERK5 activation. However, to our knowledge, the metabolic remodeling to FAO by metastatic cells has not yet been linked to immune cell recognition (64).

## 6 ERK5 is implicated in tumor immune surveillance

The interaction between different types of cells determines cancer growth and progression in the tumor microenvironment (TME). In this context, ERK5 is involved in several processes that stabilize the TME by immune system effector mechanisms, particularly those targeting tumor cells and pro-tumorigenic cell populations (12). Decreasing ERK5 in malignant mesothelioma cells injected into mice resulted in a significant reduction of tumor growth, due to downregulation of the pro-inflammatory cytokine CCL5/RANTES and other angiogenesis-related factors (69). Additionally, ERK5 is a crucial regulator of the pleiotropic cytokine IL-6 in various lung cancer cell lines and cancer-associated fibroblasts (CAFs), which modulate IL-12p70 secretion by dendritic cells (DCs), ultimately impairing their ability to induce a type 1 anti-tumor immune response (70). However, the precise mechanism by which ERK5 regulates IL-6 secretion remains to be elucidated. Furthermore, CAFs can also induce ERK5 phosphorylation and activation in colorectal cancer cells, leading to increased PD-L1 expression and promoting cell proliferation and survival. Nonetheless, *in vivo* experiments are needed to confirm these findings and clarify the connection between ERK5 and PD-L1 in the TME context (71). Tumor-associated macrophages (TAMs), derived from circulating monocytes recruited to the tumor site, exhibit an angiogenic and immunosuppressive phenotype that contributes to tumor growth and cancer progression (72, 73). ERK5 is overexpressed in TAMs from various tumor types, including breast, pancreatic, and lung cancer (74). Notably, tumor growth was reduced in mice with TAMs deficient in ERK5 signaling, primarily due to impaired activation of STAT3 signaling. These TAMs were unable to support tumor growth and angiogenesis *in vivo* (75), suggesting that the relevance of the ERK5/STAT3 axis is not limited to migrating tumor cells and EMT processes.

Moreover, ERK5 influences the expression of molecules involved in immune cell recognition (17, 76, 77), such as MHC-I (76, 77). Interestingly, forcing tumor cells to undergo respiration, such as through dichloroacetate (DCA) treatment, induces ERK5 expression and, consequently, MHC-I expression (17). Metabolism-regulated MHC-I expression can either enhance or reduce tumor cell recognition by cytotoxic lymphocytes (78). Other metabolic drugs also affect tumor cell immune surveillance. For instance, metformin, which inhibits glycolysis, induces MHC-I expression (79), facilitating recognition by natural killer (NK) cells and cytotoxic T lymphocytes (CTLs). This effect is mediated through the expression of Natural Killer G2-D (NKG2D) ligands (NKG2DL) and, primarily, intercellular adhesion molecule-1 (ICAM-1). Similarly, DCA induces NKG2DL and ICAM-1 expression, but this effect requires the presence of wild-type p53 (80). Finally, ERK5 modulates cancer cell sensitivity to extrinsic apoptosis triggered by death-receptor agonists (81), a mechanism used by cytotoxic lymphocytes to eliminate target cells.

Altogether, these observations suggest that ERK5 regulates multiple aspects of cell interactions within the TME, influencing immune recognition of tumor cells, immune cell activation, and the strength of the anti-tumor response.

## 7 Natural killer cells mediate metastasis immune surveillance

This subject has recently been reviewed (82, 83), and we will provide here only a summary of the field. EMT is one of the fundamental steps in the detachment of cancer cells from the primary tumor to initiate the metastatic process (84). During this process, metastatic cells are extensively exposed to immune cells, primarily cytotoxic lymphocytes, which patrol the blood and lymph. NK cells rapidly encounter metastatic tumor cells, leading to ERK activation and subsequent apoptosis of metastatic tumor cells (85).

NK cells play a key role in metastasis-specific immunosurveillance (86–89), and their numbers and activity correlate with the quantity of circulating tumor cells and metastases in various cancers (83, 90). Patients with different cancers who exhibit low levels of peripheral or infiltrating NK cells at tumor sites tend to have a higher number of metastatic lesions (82).

NK cells identify target cells by recognizing ligands on their plasma membranes. Although the mechanism by which NK cells recognize and kill metastatic cells is not fully understood, metastatic cells frequently lose MHC-I, which reduces inhibitory signaling and makes them more susceptible to NK cell-mediated killing (82). E-Cad, an epithelial marker, is downregulated by the transcriptional repressor Snail, which is induced during the EMT. In a model of TGFβ-induced EMT, E-Cad is recognized by Killer Cell Lectin-Like Receptor G1 (KLRG1), an inhibitory receptor on NK cells. Meanwhile, CADM1 is recognized by CRTAM, a receptor that activates NK cells (88). Thus, by decreasing E-Cad and increasing CADM1, metastatic cells can be more effectively recognized by NK cells. This partially explains metastatic cell recognition; however, losing the inhibitory interaction between E-Cad and KLRG1 alone is insufficient to reactivate NK cells’ cytotoxic functions (88).

Other ligands of NK cell activating receptors also increase during EMT such as PVR, UL16 binding protein2 (ULBP2), ULBP4, MHC class I polypeptide–related sequences A (MICA) and B (MICB), along with a decrease in the inhibitory ligand MHC-I (88, 91). The activating NKG2D receptor binds to MICA and MICB and various ULBPs, leading to target killing (92). In contrast, the inhibitory Killer-cell immunoglobulin-like receptor (KIR) recognizes MHC-I (93). Recognition of these ligands is crucial for NK cells’ anti-metastatic functions, and enhancing NK cell cytotoxicity within tissues can further strengthen the elimination of metastatic cells (94). Therefore, regardless of the ligands expressed on metastatic cells, strategies that boost NK cell function can improve their immune surveillance capabilities in this context.

NK cells are likely more effective at recognizing and/or eliminating individual metastatic cells or small metastatic clusters (83, 95). In contrast, circulating tumor cell (CTC) clusters are less sensitive to NK cells (83). Although large clusters exhibit reduced expression of NK cell-activating ligands (95), which can explain their decreased sensitivity to NK cells, the size of the cluster may also inhibit NK cell infiltration. For instance, clusters show elevated expression of epithelial and cell–cell adhesion genes (95), which likely prevents NK cell infiltration.

Another notable connection between ERK5 and the tumor microenvironment is its role in promoting EMT and a FAO/OXPHOS metabolism (10, 11), which may lead to increased extracellular reactive oxygen species (ROS), that suppress NK cell function. However, the presence of IL-15 protects NK cells from the detrimental effects of ROS (96), suggesting that activated NK cells may be shielded from ROS-induced damage.

## 8 Is ERK5 the link between metastasis and their recognition by NK cells?

We have described that ERK5 regulates immune cell recognition, EMT and FAO. Based on this, we hypothesized that ERK5 signaling could help explain why metastatic cells are promptly recognized by NK cells. Our unpublished findings show that rewiring cancer cell metabolism towards FAO using DCA activates ERK5, induces EMT gene expression (both *in vitro* and *in vivo*), and promotes tumor cell migration and invasion (97). Furthermore, DCA triggered the expression of a specific pattern of ligands in tumor cells, which facilitated NK cell recognition. As a result, NK cells infiltrated 3D tumor spheroids and exerted their cytotoxic effects (97). FAO drives EMT and the metastatic process, at least partially, through ERK5 activation. During EMT, various mechanisms could induce the expression of ligands recognized by NK cells, thereby enhancing immune surveillance and partially explaining the inefficiency of the metastatic process (86, 90). Thus, EMT and increased immune surveillance are linked, representing an Achilles’ heel for metastatic cells by limiting their ability to colonize new environments.

This insight could be leveraged in clinical settings by using specific metabolic drugs to promote the expression of NK cell-activating ligands on the tumor cell surface (18, 64), such as Intercellular Adhesion Molecule-1 (ICAM-1) or NKG2DL (80, 98), in combination with cytotoxic lymphocyte-based therapies to treat metastases.

ERK5 inhibition has also been shown to induce cellular senescence, making senescent cells susceptible to elimination by natural killer (NK) cells (68). This suggests that ERK5 may play a role not only in NK cell recognition of metastatic cells but also in the detection of senescent cells. These findings highlight a broader connection between ERK5 expression on target cells and NK cell-mediated immune surveillance.

## 9 Targeting ERK5 roles to fight metastasis

Although we will not detail ERK5 inhibitors (ERK5i) here, it is important to note that inhibition of ERK5 kinase activity does not necessarily block overall ERK5 function, given its kinase-independent roles (99, 100). Some ERK5i even paradoxically enhance C-terminal transactivation domain (TAD) activity and nuclear localization (101). This prompted the development of ERK5 degraders, such as the PROTAC INY-06-061, which selectively eliminates ERK5 protein. However, acute degradation failed to reproduce the anti-proliferative and anti-inflammatory effects observed with genetic ablation or earlier inhibitors (102). These findings dampened enthusiasm for ERK5 as an oncology target (102), suggesting that highly selective ERK5i lack intrinsic cytotoxicity, and should lack important *in vivo* toxic effects.

The ERK5 connections described here, particularly its role in immune surveillance, could represent interesting pharmaceutical targets to enhance immune cell activity. For example, immune checkpoint inhibitors (ICIs) are not directly cytotoxic but promote tumor cell destruction by the immune system, albeit with significant adverse events (103). Similarly, interfering with ERK5-mediated mechanisms could improve cancer treatment, especially against metastasis. Furthermore, engraftment of allogeneic, pre-activated NK cells may also prevent metastatic cell dissemination by eliminating them in the bloodstream. These approaches could provide new therapeutic options for patients at risk.

## 10 Conclusion

The spread of metastatic cells to distant locations is the leading cause of cancer-related deaths. NK cell-mediated immune surveillance plays a crucial role in regulating metastatic dissemination and represents a promising therapeutic target (90). Unveiling the mechanisms that link ERK5 to FAO and/or the expression of immune cell ligands and NK cell recognition could open new avenues for clinical treatments. The survival of metastatic cells depends on their ability to reprogram metabolism in order to colonize new environments, a process that involves ERK5. However, ERK5 activation concurrently induces the expression of a ligand repertoire recognized by NK cells, ultimately resulting in the elimination of these metastatic cells: we propose that these dual functions of ERK5 constitute a pharmacologically targetable Achilles’ heel of metastatic cells.
